# The Process of Developing an Intervention to Increase Awareness of Cardiovascular Risk for Persons With Type 2 Diabetes: Co-Creation Study

**DOI:** 10.2196/85748

**Published:** 2026-04-23

**Authors:** Anna-Lena Stenlund, Karin Hellström Ängerud, Mikael Lilja, Julia Otten, Lena Jutterström

**Affiliations:** 1 Department of Nursing, Umeå University, Campus Skellefteå, Bockholmsvägen 23, Umeå, Skellefteå, SE-931 87, Sweden, +46 910787214; 2 Department of Public Health and Clinical Medicine, Umeå University, Umeå, Sweden

**Keywords:** co-creation, participatory action research, PAR, intervention development, type 2 diabetes, cardiovascular risk: risk awareness

## Abstract

**Background:**

Many persons with type 2 diabetes (T2D) lack risk awareness or underestimate their cardiovascular risk. Although health care professionals in primary health care strive to implement risk-awareness strategies for cardiovascular risk, persons with T2D report a lack of meaningful dialogue with health care professionals. Co-creation is grounded in participatory action research and involves participants as equal partners across all stages of a project. This study describes the development of an intervention to increase cardiovascular risk awareness in people with T2D.

**Objective:**

This study aims to describe the co-creation process of developing an intervention to increase awareness of cardiovascular risk in persons with T2D.

**Methods:**

A co-creative design was used to develop an intervention following a participatory action research framework. Four workshops with persons with T2D, diabetes specialist nurses, and physicians in primary health care explored communication about cardiovascular risk, co-identified needs, co-designed solutions, tested prototypes, and redefined and retested the content of the intervention. The data were analyzed using reflexive thematic analysis.

**Results:**

The analysis identified 4 themes: co-define: taking the person’s voice into account; co-design: problem-solving and generating ideas; prototype and test: drafting intervention proposals; and redefine and retest: reviewing suggested interventions. The workshop discussions highlighted the need for new interventions, including a risk assessment tool, a patient handbook, material to prompt reflection, and a web education for specialist diabetes nurses.

**Conclusions:**

This study demonstrates the value of co-creation, which was used to develop an intervention to enhance cardiovascular risk awareness in persons with T2D. Diabetes specialist nurses need to explore patients’ perceptions of risk and provide space for emotional responses. The web education is intended to strengthen the person-centered approach of diabetes specialist nurses, the patient handbook encourages reflection and dialogue on personal risk, and the risk assessment tool visualizes individual risk. These components may contribute to increased awareness of cardiovascular risk.

## Introduction

### Cardiovascular Risk in Type 2 Diabetes

Persons with type 2 diabetes (T2D) face an elevated risk of micro- and macrovascular complications, including a two- to four-times increased likelihood of developing cardiovascular diseases such as myocardial infarction and stroke [1]. Early detection of T2D and timely optimization of hemoglobin A1c (HbA_1c_) and other risk factors are crucial for preventing cardiovascular events [2]. Despite this, not all persons with T2D reach treatment goals [3]. The prevention of cardiovascular risk involves addressing behavioral risk factors, such as regular exercise, healthy eating, smoking cessation, and medical treatment [4]; effective risk communication reduces the overall risk and enhances persons’ self-perceived risk motivation [5]. Health care professionals (HCPs) in primary health care are expected to provide person-centered communication of cardiovascular risk; however, research shows that risk communication is often one-sided, with HCPs mainly informing rather than engaging with persons [6].

### Background

Many persons with T2D lack risk awareness or underestimate their cardiovascular risk, particularly in the absence of symptoms [7]. Although the link between T2D and cardiovascular risk is well established, effective risk communication regarding myocardial infarction and stroke remains a challenge [8]. Person-centered care can improve risk awareness and self-management. Interventions building on the self-determination theory also improve health behavior for self-management and motivation [6]. Interventions based on self-determination theory can improve changes in health behavior and autonomy motivation for self-management (ie, 9) and can significantly reduce cardiovascular risk [9]. However, HCPs find it difficult to perform person-centered care in clinical practice [10].

HCPs in primary health care should strive to implement risk-awareness strategies for cardiovascular risk in T2D; however, persons with T2D report a lack of meaningful dialogue with HCPs [8] and express a desire for more personalized, participatory communication. Risk communication must be individual-specific enough to make a person with T2D perceive their own risk without inducing fear or anxiety but rather fostering understanding and motivation [11]. Trust and shared decision-making have been highlighted as important to effective risk communication and patient motivation [12]. While HCPs are expected to communicate cardiovascular risk to persons with T2D [7], there is limited guidance on how to do so effectively; therefore, more research in this area is needed.

Co-creation has emerged as a promising approach to intervention development and increasing participation and involvement in health care [13]. Co-creation is rooted in participatory action research (PAR) and involves participants as equal partners throughout all stages of a project [14]. It facilitates joint problem identification, shared understanding, and context-specific solutions [15] and enhances the effective development of interventions [14]. Co-creation design uses methods and strategies to identify problems, improve user experience, and adapt functionality [15]. Studies within PAR have shown that when participants are involved in shaping the design of an intervention, they are more likely to adopt it in clinical practice [16].

Effective interventions should enhance risk awareness, strengthen motivation, and support behavior change related to elevated cardiovascular risk. This requires incorporating diverse perspectives and ensuring that interventions are both theoretically sound and practically applicable [14]. Interventions that raise awareness of cardiovascular complications, while also addressing attitudes and self-management, benefit not only persons with T2D but also the health care system [13].

Our previous qualitative studies involving persons with T2D and HCPs in primary health care indicate that cardiovascular risks are not communicated sufficiently and that the risk communication that does occur during clinical consultations is often ineffective for persons with T2D [17]. There is a need for more interventions developed using co-creation to improve risk communication and thereby enhance risk awareness among persons with T2D. This study aims to describe the process of developing an intervention intended to strengthen the awareness of persons with T2D of their cardiovascular risk and foster more effective self-management of the condition.

### Aim of the Study

This study aims to describe the co-creation process of developing an intervention with the aim of increasing awareness of cardiovascular risk in persons with T2D.

## Methods

### Design

A co-creation study design within a PAR framework [13,14] was used.

### Study Setting and Recruitment

This study was carried out as a series of workshops that followed a PAR framework and involved persons with T2D and HCPs working in primary health care in northern Sweden. To recruit participants, posters were distributed to primary health care units. No one answered; therefore, diabetes specialist nurses were contacted in order to suggest participants in the form of both persons with T2D and HCPs. Thereafter, a snowball sampling was conducted for persons with T2D, diabetes specialist nurses, and physicians who fulfilled the inclusion criteria. The inclusion criteria for persons with T2D were a diagnosis of T2D, being 18 years or older, and being Swedish-speaking. The HCPs needed to be diabetes specialist nurses or physicians working with diabetes in primary health care.

In total, 10 persons with T2D (median age 68.5 y, range 52‐79) and 5 HCPs (median age 53 y, range 32‐59) participated (Table 1). The HCPs were 3 diabetes specialist nurses and 2 physicians, with working experience in primary health care ranging from 2 to 20 years (Table 2). Before the workshops, the participants received information about the study both orally and in writing and had the opportunity to ask questions. Written consent was obtained from all the participants. Workshops 1 and 2 included persons living with T2D and 2 researchers. Workshops 3 and 4 included diabetes specialist nurses, physicians, and 2 researchers. Thus, a different set of participants attended each workshop.

**Table 1.** Demographic characteristics of persons with type 2 diabetes (n=10).

Characteristics	Values
Diabetes duration (y), median (IQR)	13.5 (2‐21)
Age (y), median (IQR)	68.5 (52‐79)
Gender, n	
Female	5
Male	5
Education level	
Primary school level or upper secondary school level	6
University or higher education level	4
Cardiovascular history, n	
Yes	2
No	8
Treatment for high blood pressure or blood lipids, n	
Yes	8
No	2
Smoking, n	
Yes	0
No	10

**Table 2.** Demographic characteristics of the health care professionals (n=5).

Characteristic	Participants
Diabetes specialist nurses	3
Physicians	2
Professional experience (y), range	2‐20
Age (y), median (range)	53 (32‐59)
Gender, n	
Female	4
Male	1
Digital literacy, n	
Yes	5
No	0

### Data Collection

#### Overview

**Figure 1.**
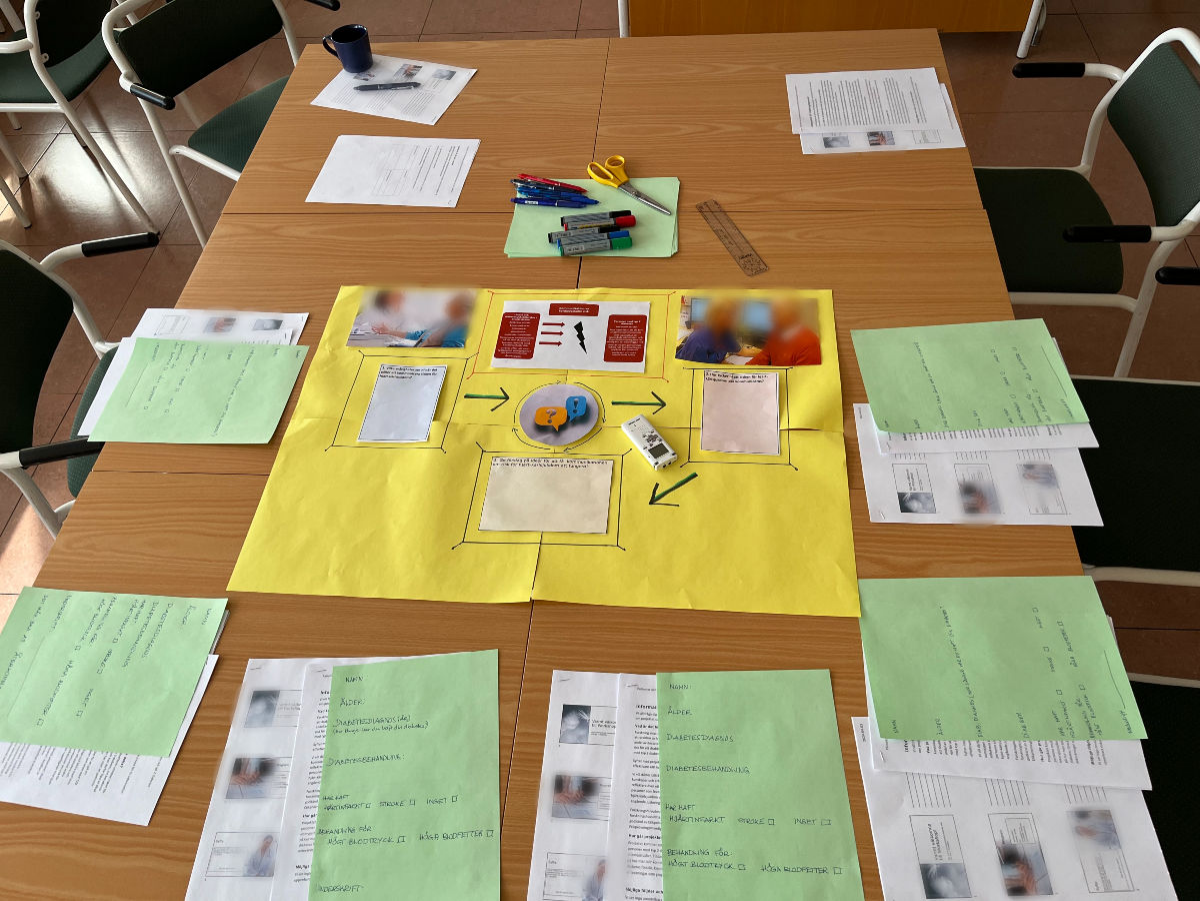
Examples of probes and toolkits for the participants of workshops 1 and 2.

Before the workshops, the researchers conducted a review of the results of both their own previous studies and those of other researchers. They planned and designed the structure and flow of the sessions and prepared probes, toolkits, the agenda, activities, and supporting materials. The workshop materials included presentations, images, worksheets, and scenario-based exercises. Figure 1 shows examples of probes and toolkits for the participants of workshops 1 and 2. In workshops 3 and 4, PowerPoint material was used to share the findings from previous workshops.

Data collection was conducted at the workshops involving persons with T2D and 2 researchers (workshop 1, n=8; workshop 2, n=6) and the HCPs and 2 researchers (workshop 3, n=4; workshop 4, n=5). The workshop discussions included 2 researchers, who facilitated the workshops and produced field notes. The workshops were held in Swedish, audio-recorded, and transcribed verbatim and analyzed using reflexive thematic analysis. Each workshop lasted between 90 and 120 minutes. Workshops 1 and 2 were held in person at the university; workshops 3 and 4 were held online. All workshops were held between May and November 2024.

#### The Co-Creation Process Within a PAR Framework

PAR is a qualitative methodology where participants actively engage in the research process [18]. It values the knowledge and experiences of participants, makes use of democratic processes for positive change, and emphasizes action [19]. The process involves iterative cycles of relationship-building and colearning to identify problems, design solutions, and reflect on actions over the course of at least three cycles [20]. Each cycle consists of 4 stages: observe, reflect, plan, and act [16]. The observation stage involves clarifying and identifying problems, needs, and barriers. In the reflection stage, the participants analyze and reflect, listen to different perspectives, and share ideas and experiences. The planning stage involves clarifying and drafting proposals and developing an action plan. The action stage involves visualizing the ideas, shaping and revising the intervention [16]. One review found that PAR is frequently applied in several phases of the research process such as data collection, research design, and recruitment and is grounded in the real-world experiences of participants [18]. In this study, 4 cycles were conducted. Persons with T2D and HCPs were actively involved in data collection, interpretation of findings, co-defining, co-designing, prototyping, and testing, as well as redefining and retesting the intervention.

**Figure 2.**
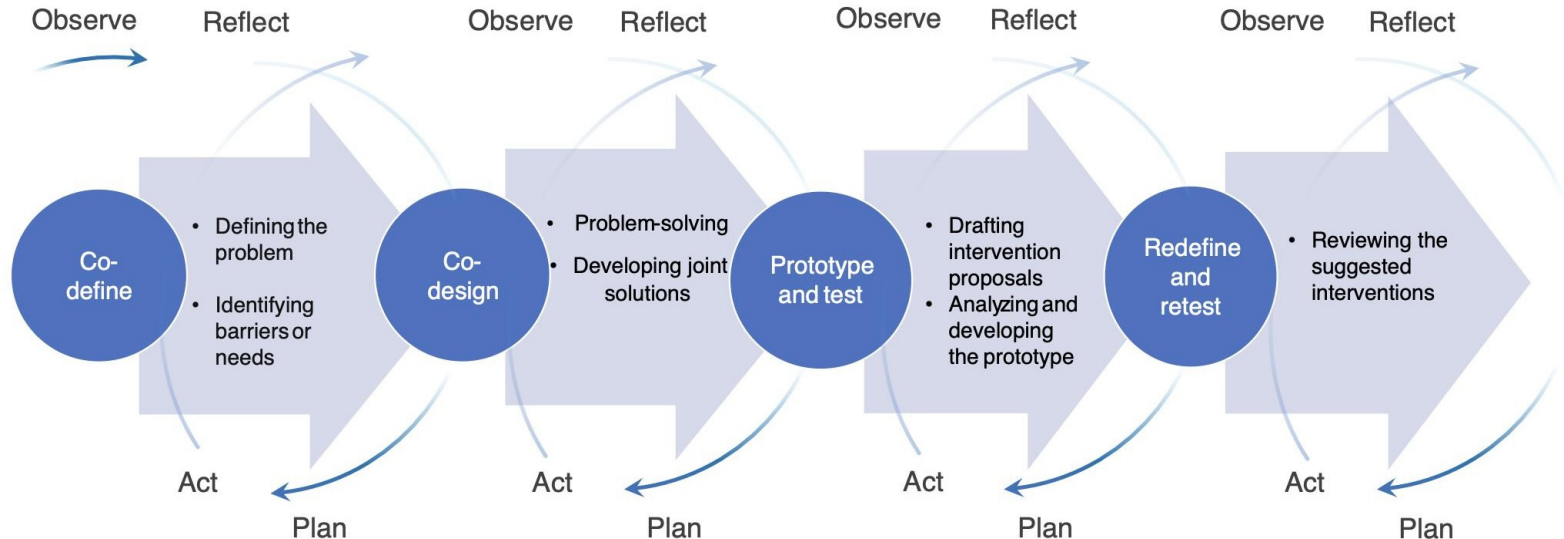
An overview of how the participatory action research (PAR) process proceeded and how the results evolved progressively over the course of the 4 co-creation cycles. The figure is influenced by previous research [16,19-21].

Figure 2 illustrates the co-creation process of the PAR framework followed in this study: co-define, co-design, prototype and test, and redefine and retest, influenced by previous studies [16,19-21]. The co-define stage included the identification of barriers and needs and problem formulation. The co-design stage included solution development, and the prototyping and testing stage involved evaluating and forming intervention prototypes. The redefine and retest stages included improvement and evaluation and revising the intervention (Figure 2) [16].

The facilitators (researchers) in this study did not propose ideas; instead, they encouraged the participants to share perspectives, suggest strategies, and co-create solutions. Based on these discussions, the researchers developed materials to facilitate risk communication and strengthen risk awareness among persons with T2D.

### Data Analysis

**Figure 3.**
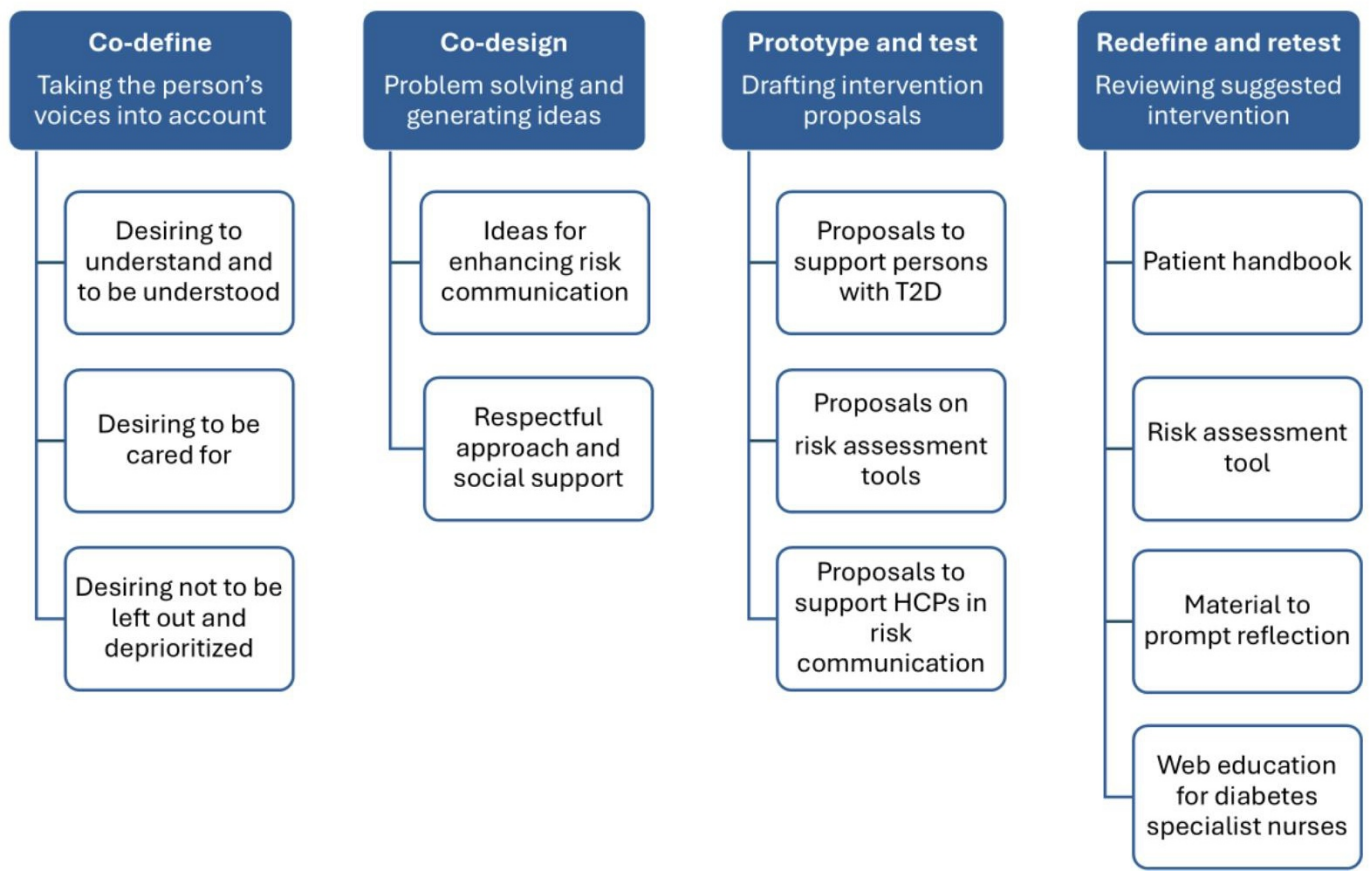
Overview of the themes and subthemes. HCP: health care professional; T2D: type 2 diabetes.

Reflexive thematic analysis was conducted to analyze the workshop transcripts, following the steps outlined by Braun and Clarke [22]. To become *familiar with the data,* the first author read the verbatim transcripts (58,596 words) and field notes multiple times, making notes and marking sentences and paragraphs that were relevant to the aim of the study [22]. The initial coding was carried out inductively by the first author using MAXQDA 2022 to facilitate the organization, sorting, and coding of the data. A manifest analysis approach was used, which involved highlighting sections of text, interpreting the obvious in the text, identifying relevant phrases and sentences, developing codes (1123 codes) to describe their content, and sorting the codes into groups. Paraphrasing and marginal notes were incorporated to preserve contextual relevance and support the analytical process when developing subthemes. Thereafter, the first author incorporated latent thematic analysis to explore the deeper meanings of the text. In this study, guided by the principles of PAR, the analytical process shifted from an inductive to a deductive approach when *generating themes*. Themes were defined through iterative collaborative reflection (ALS, LJ), in which the researchers identified patterns in the material and grouped them into themes and subthemes based on the content (Figure 3). The validity and reliability of the themes were ensured through the development of a thematic map that illustrated the relationships among codes, themes, and subthemes (Multimedia Appendix 1). The themes were then defined and named through iterative discussions between the 3 authors until consensus was reached. Subsequently, the entire research group met to agree on the final themes (ALS, LJ, KHÄ, JO, ML) (Multimedia Appendix 1).

### Ethical Considerations

Ethical approval was granted by the Swedish Ethical Review Authority (2018-12-11; Dnr 2023-06037-02). The clinical director and department heads of the primary health care centers approved the study. The participants were informed verbally and in writing about the study’s aim, their participation and right to withdraw, and confidentiality. Written informed consent was obtained from all participants and they were informed that participation was voluntary and that they could withdraw from the study at any time. In addition, informed consent was obtained from the participants to use their images for publication purposes. Furthermore, they were guaranteed confidentiality, with the results presented at group level to avoid identification and quotations deidentified. The participants did not receive compensation.

### Rigor and Reflexivity

The reflexive thematic analysis reporting guidelines were used to ensure the rigor, coherence, and reflexive openness of the study (Checklist 1) [23]. The co-creation design ensured credibility [24]. The research process was transparently documented, with data collection, analysis, and interpretation detailed. Trustworthiness was enhanced by clearly describing the methodology and maintaining reflexivity to minimize bias. The researchers’ nursing and teaching experiences enabled them to ask relevant questions and helped build trust with the participants. To mitigate bias, the researchers adopted a reflexive stance, systematically examining their assumptions and preconceptions. This reflexivity was essential to uphold ethical rigor and enhance the trustworthiness of the data collection process. The researchers came from diverse backgrounds and held regular meetings to critically reflect on the data. Confirmability was maintained through a systematic coding process and rigorous data analysis, which ensured that the findings were grounded in the accounts of the participants. Credibility was strengthened by building trust, clarifying goals, and fostering inclusive dialogue with the participants. Transferability was supported by thorough documentation of context, including participant demographics (eg, age, disease duration, previous cardiovascular events) and setting.

## Results

### Themes and Subthemes

The analysis resulted in 4 themes and 12 subthemes, wherein the participants identified challenges, generated ideas, prototyped intervention options, and evaluated these options for cardiovascular risk communication (Figure 3). The co-creation process and the findings of the thematic analysis are presented under 4 identified themes: co-define: taking the person’s voice into account; co-design: problem-solving and generating ideas; prototype and test: drafting intervention proposals; and redefine and retest: reviewing suggested interventions.

### Co-Define: Taking the Person’s Voice Into Account

#### Overview

In cycle 1, the focus was on identifying communication barriers and needs from the perspectives of the persons with T2D. The participants’ actions included discussing problems, challenges, and needs and sharing experiences. The researchers began every workshop by introducing the topic and purpose of that workshop, as well as discussing previous research and workshops. The participants were encouraged to discuss the findings of previous research, including our previous interview studies with persons with T2D and diabetes nurses and physicians.

#### Results of the Thematic Analysis

##### Desiring to Understand and to Be Understood

Persons with T2D want HCPs to communicate cardiovascular risk in various ways, and in a manner that they can understand, to ensure clarity and comprehension. The participants stated that medical jargon and complex terminology should be avoided, and clear and simple language should be prioritized:

Don’t say cardiovascular risk; say myocardial infarction and stroke.

[Participant, cycle 1]

The participants described not fully understanding their risks of myocardial infarction and stroke, and many claimed that they had never heard that T2D could lead to myocardial infarction and stroke. The participants expressed a desire for clarity regarding the meaning of test results, such as explaining HbA_1c_ and low-density lipoprotein cholesterol values, and how these impact cardiovascular risk.

Just this thing about what could happen if I’ve had this for so long – am I having a myocardial infarction? Is a stroke coming? But no one has told me about that.

[Participant, cycle 2]

The participants shared the belief that HCPs were too vague when discussing these risks, and the results indicate that the participants believe that HCPs avoid discussing risks and even downplay them because they are afraid of scaring patients or handling emotional reactions.

Yes, and I also think it’s too sensitive for many people, even within healthcare, to talk about this. Yet, it’s something we all know – that we’re all going to die someday.

[Participant, cycle 1]

Many of the participants expressed a preference for receiving guidance in both written and verbal form, to reinforce their understanding of their cardiovascular risk and help them with the self-management activities involved in reducing it. The participants expressed the need for simple, relevant materials and guidance regarding where they could independently explore information suited to their individual needs.

Sit and listen first, but then I would like to have it written down on paper – yes, almost. I mean, clear communication that I can go back to and read later.

[Participant, cycle 1]

##### Desiring to Be Cared For

The participants described the importance of their interactions with HCPs, highlighting these encounters as a crucial aspect of their diabetes care. However, a recurring concern was the perceived lack of curiosity, interest, and engagement from HCPs. The persons with T2D stated that HCPs should show more interest in them as persons and ask more questions to help them feel more involved in their own care. The participants highlighted the need for undivided attention, emotional presence on the part of HCPs, and meaningful dialogue, as well as a communication style that conveys hope, encouragement, and care without blame. Persons with T2D described experiences of inconsistent communication and perceived nonchalance, which contributed to feelings of being dismissed or undervalued in the health care encounter. The participants described wanting to feel truly heard and cared for:

In that moment, I want to feel like you care only about me and that she should go through what has happened previously – what my blood sugar levels were like back then – and also explain the results of the tests I took a few days earlier. And maybe answer questions like: how does this work? Is it good or bad, or somewhere in between? And what can I do?

[Participant, cycle 2]

The results show that persons with T2D would like to feel trust and understanding during meetings with HCPs but are often not even asked how they are feeling:

I have never experienced a diabetes nurse asking anything like, how are you feeling? Are you affected by seasonal depression? I mean, she could ask anything … but never.

[Participant, cycle 1]

Persons with T2D expressed that stress and the emotional burden of managing diabetes further complicate risk communication, noting that these factors can reduce their attention and contribute to feeling overwhelmed. Furthermore, the results revealed that this emotional topic was seldom addressed in risk communication and that HCPs tended to follow their own agendas.

##### Desiring Not to Be Left Out and Deprioritized

Insufficient follow-ups, limited continuity of care with HCPs, time constraints in health care, and a sense that patients were merely being “checked off” rather than supported were described. The results indicate that HCPs control and determine the content of discussions during diabetes appointments, contributing to persons with T2D feeling excluded and perceiving that decisions are made over their heads. This perceived nonchalance reinforced feelings of being dismissed or undervalued during health care encounters. As one participant expressed:

Just for a moment, and the moment is decided by her, how much time she has, and then we don’t talk about anything else. We switch to the “red light” at the same time; it feels like you said – I’m here to find out something. I go there with the suspicion that maybe I’ll die tomorrow, or how else should I feel? I don’t know anything else.

[Participant, cycle 2]

The results illustrate that persons with T2D experience a lack of structure in health care and feel that they are not prioritized when their needs are not addressed. The participants described negative attitudes and stigma relating to diabetes on the part of HCPs, and these influence their health care experiences and further contribute to their sense of being part of a deprioritized group.

It’s important not to feel that the T2D patient group is deprioritized or placed after all other patient groups.

[Participant, cycle 2]

Persons with T2D described feeling that they must take responsibility for their care on their own. Several participants expressed dissatisfaction with having to remind HCPs about the regular collection of blood samples:

There is a lot of engagement required from the patient. You have to remind them about testing and contact with the diabetes nurse varies. Sometimes, you have to beg to get tests done, and most of the time, you have to find out about risks yourself. They don’t ask about family history.

[Participant, cycle 2]

The results revealed that persons with T2D also want diabetes specialist nurses to acknowledge the emotional aspects during consultations.

### Co-Design: Problem-Solving and Generating Ideas

#### Overview

The co-design stage involved the development of ideas and joint solutions, which were based on previous studies and the results of the first cycle of inquiry. Several suggestions for improving risk awareness in T2D were presented. The findings indicate that effective risk communication should be individualized, in verbal and written form, be direct rather than vague, be accessible, and easy for persons with T2D to refer to.

#### Results of the Thematic Analysis

##### Ideas for Enhancing Risk Communication

To address barriers to risk communication, both persons with T2D and HCPs highlighted the need to acknowledge individual perspectives on risk. The results highlighted the importance of combining verbal and written communication, such as note-taking and providing clear summaries, to support the understanding of patients. The results of cycle 3 suggest that HCPs see the patient group as diverse and recognize that individuals require different approaches that are adapted based on preferences and needs. Relying solely on digital communication is not suitable, as many people with T2D are older and not digitally literate. According to the results, using paper-based documents is a helpful option.

One suggestion for improving risk communication was to encourage individuals with T2D to reflect on and articulate their perceptions of their own cardiovascular risk. Questions that prompt reflection were considered to be essential, including those that explore patients’ understanding of cardiovascular risk, self-management activities, emotional responses, and future expectations, as well as the role of family support.

In cycle 3, a risk assessment tool was proposed to support follow-ups and help individuals with T2D to monitor the progression of their cardiovascular risk over time. The participants emphasized the importance of providing patients with laboratory values related to their own risk, along with asking them exploratory questions to explore how they perceive that risk.

An idea that emerged involved illustrating cardiovascular risk levels using colors to enhance understanding. One suggestion was to develop a patient handbook that includes essential information and material that is intended to prompt reflection, to improve the person’s risk awareness.

A kind of book that the patient takes with them. Maybe they would have tasks in the book, such as: find out this for the next time you meet with your diabetes nurse or doctor. There are things you need to understand and know, and that’s what we should focus on.

[Participant, cycle 3]

##### Respectful Approach and Social Support

The results showed the importance of social support and a sense of equality during health care interactions. The HCPs asserted that building trust, fostering engagement, and encouraging change were essential for persons with T2D to feel comfortable opening up and sharing their thoughts and feelings about their risk. The results showed that the HCPs feel that it is important to be professional, with the aim of reducing inequality in interactions. This involves not underestimating individuals with T2D, treating them with respect, instilling hope, allowing questions, avoiding blame, and demonstrating genuine care and trustworthiness. Addressing power imbalances was posited as an aspect to improve the interaction between persons with T2D and HCPs. Social support and peer interaction were also highlighted as valuable components, with suggestions of group meetings and family-inclusive consultations to improve risk awareness. Some HCPs argued that a relative accompanying a person with T2D to an appointment meant that information could be discussed further at home:

If you [the patient] have the opportunity to have someone from your close family or a partner with you, who will take notes, it’s really helpful.

[Participant, cycle 3]

### Prototype and Test: Drafting Intervention Proposals

#### Overview

At the beginning of cycle 3, the researchers presented previous discussions, ideas, and drafts from the co-define and co-design stages. The HCPs evaluated the discussions and scenarios used during workshops 1 and 2 with persons with T2D by analyzing, reflecting on, and discussing difficulties and strategies relating to, and the structure of interventions. The HCPs were encouraged to share the challenges they face in communicating cardiovascular risk, propose strategies, and develop the design and structure of the prototype intervention. The workshops were structured to encourage collaboration and facilitate the gathering of valuable insights regarding the communication of cardiovascular risk.

#### Results of the Thematic Analysis

The proposals identified as important elements of an intervention to increase risk awareness are described below.

##### Proposals to Support Persons With T2D

Based on the results of cycle 3, ideas emerged regarding a patient handbook to support persons with T2D. This was intended to help them reflect on and understand their risk of myocardial infarction and stroke individually, with family members, and in dialogue with diabetes specialist nurses and physicians, emphasizing person-centered care.

Having a small book, where you write down your questions, so you can bring them to the meeting we’re going to have, because you’ll never remember everything. We’ll talk about so many things, and then you’ll remember the things you really want to know.

[Participant, cycle 3]

Another suggestion was to develop discussion materials that could facilitate risk communication and encourage a progression in the discussions – moving forward, rather than repeating the same information. It was expressed that diabetes specialist nurses and physicians need to promote patient involvement, actively listen to the issues or topics that persons with T2D wish to discuss, and demonstrate genuine interest in the patient, rather than following their own agenda.

And then the patient can go back and say: oh, right, we talked about this last time. What was it again? And then there could be some sort of reference so that as a patient, you can go in here and read a bit more about it. It doesn’t need to be anything overly complicated.

[Participant, cycle 3]

The results highlighted the use of materials to provoke reflection, to help persons with T2D assess their level of cardiovascular risk. Such materials were felt to help the persons to reflect independently, with family members, or in collaboration with health care professionals, all of which serve to enhance their understanding of the risks involved.

One should be able to include questions that the patient, on their own or together with someone, can reflect on.

[Participant, cycle 3]

At the same time, it was noted that a book may not provide suitable support to all persons with T2D. One physician noted the importance of integrating the personal handbook into visits, but that not all persons may be ready or interested in engaging with such exercises.

##### Proposals on Risk Assessment Tool

During cycle 3, the participants suggested using visual tools, such as color-coded images to provide information regarding different levels of risk for myocardial infarction and stroke (low, medium, high). However, some of the participants described red as an “angry” color, prompting discussions about how visual pathways can be used to illustrate steps toward lower risk levels and improved motivation for self-management activities.

It should visually show something like, you’re at high risk, but the red feels a bit angry to me. Could we create pathways forwards from the red? I mean, this might sound childish, but maybe we could make images that show how to move on from there …

[Participant, cycle 3]

##### Proposals to Support HCPs in Risk Communication

One suggestion that emerged from the co-creation process was that HCPs need to improve their ability to adopt a person-centered approach when communicating cardiovascular risk. Therefore, a web education was developed that addresses risk communication. Intervention ideas from the co-creation process were used in the development of the web-based education, which was intended for diabetes specialist nurses and focuses on important aspects of person-centered risk communication and exploring individual risk. The web-based education explains the aim of the patient handbook and how to use risk assessment tools and reflective questions.

The networking sessions we have each semester are more about receiving communication on current topics, like something about test strips, procurements, and such. Perhaps what we need is more about how to use tools and exchange ideas on how to work, how to communicate, and how to reach these persons. What am I doing wrong?

[Participant, cycle 3]

### Redefine and Retest: Reviewing Suggested Interventions

#### Overview

The researchers analyzed results from previous workshops and drafted intervention proposals for the participants to review during the redefine and retest stages. The Consolidated Framework for Implementation Research (CFIR) was used to develop the intervention, considering potential barriers and facilitators in risk communication (Multimedia Appendix 2). We also considered the nature of the intervention before presenting the proposal to HCPs in workshop 4 (Multimedia Appendix 1). The HCPs were encouraged to evaluate strategies and tools and suggest improvements. The proposed intervention focused on a patient handbook for persons with T2D, a risk assessment tool, to prompt reflection, and a web education to support diabetes specialist nurses. The workshop addressed how these tools could support the communication of cardiovascular risk and what additional support might be needed. Insights from this stage guided the refinement of the intervention’s content and scope.

After the development of the preliminary intervention, the researchers collected feedback from persons with T2D. They were asked to evaluate the personal handbook, the risk assessment tool, reflective material, and the web education for diabetes specialist nurses. The intervention materials were distributed to the participants, who were invited to review the content and provide feedback on its relevance, clarity, and usability. Feedback was collected either in writing via email or verbally during scheduled follow-up conversations.

#### Results of the Thematic Analysis

##### Patient Handbook

The handbook allows patients to reflect on their risk, consider strategies for managing it, and set goals to promote engagement and risk awareness. It was designed by the researchers to provide concise information on cardiovascular risk factors and practical guidance for reducing cardiovascular risk, as well as questions to prompt reflection to facilitate exploration of personal understanding of cardiovascular risk.

In workshop 4, the HCPs stated that opportunities for reflection and goal-setting could be valuable in enhancing the understanding and awareness of the risks associated with T2D for patients.

Encouraging persons to reflect on and write down their thoughts and goals can strengthen awareness of the risk of myocardial infarction and stroke.

[Participant, cycle 4]

Feedback on the patient handbook during workshop 4 indicated that it can serve as a valuable tool for reflection for people with T2D, facilitate conversations with relatives and friends, and support collaboration with diabetes specialist nurses and physicians.

It’s helpful to raise questions about what they believe regarding their abilities and how much confidence they have in themselves, so they can discuss it during the diabetes appointment.

[Participant, cycle four]

The results indicated that the patient handbook needed to be designed to encourage reflection and active participation on the part of the patient. The participants noted that, unlike traditional diabetes visits, the handbook provides an opportunity for them to consider their own perspectives and generate discussion during consultations, increasing their involvement:

No, the persons, those who receive this in their hands, of course, they can have their opinions, but it’s interesting and refreshing that they get to reflect in a way they didn’t before. We used to have these health declaration forms that they received at home before visits, and they would fill them out. This is like a different kind of thought-provoker, I think, and it could probably raise some questions that we can discuss during the visits.

[Participant, cycle 4]

##### Risk Assessment Tool

The HCPs expressed a need for a structured overview of the results of laboratory tests, along with other health parameters that are central to assessing and managing T2D. A risk assessment tool was developed by the researchers to visualize individual risk factors based on HbA_1c_, blood lipids, blood pressure, and other lifestyle-associated risk factors in diabetes, using green (low risk), yellow (moderate risk), and red (high risk) indicators during diabetes consultations. The level of risk is then discussed with the patient in relation to actions that they can take to reduce their risk and activities they can undertake as part of self-management based on personal goals.

Here is a tool to facilitate the conversation with the patient, and this is important, and this is also prioritized. I think that’s good.

[Participant, cycle 4]

The HCPs believe that the risk assessment tool can be used to engage persons with T2D and enhance their understanding of their health situations. Visualizing risk can increase motivation for self-management, thereby reducing cardiovascular risk. By asking open-ended questions about their own cardiovascular risk, persons with T2D can be encouraged to reflect on their situation, consider what actions they can take to reduce their risk, and bring attention to the emotional aspects of risk. For example: “What thoughts or feelings arise when you reflect on your risk?”

Is there anything you can do yourself to change this red number to yellow? It should be moving toward green, right?

[Participant, cycle 4]

The HCPs felt that the risk assessment tool was valuable for creating a person-centered plan that could prevent complications associated with T2D. The HCPs also argued that a color scale with numerical values would be useful for assessing risk.

Using colors to illustrate and stimulate thought processes is a great idea. It’s important to have tools that can be prioritized in the conversation.

[Participant, cycle 4]

Some HCPs believed that the severity conveyed by such visuals might be important for encouraging persons with T2D to take risks seriously. However, some HCPs believed that the severity conveyed by such visuals might be unnecessarily worrying for persons with T2D and cause them to take the risks too seriously. These varying perspectives highlight the delicate balance between providing clear, impactful communication and avoiding unnecessary fear or discomfort.

I know that I may not always do everything the right way, because I can sometimes tend to want to comfort persons when they receive a diagnosis and say “we have such good medicines now” [laughs] Like, this will go well, it’s not like before when people had to undergo amputations and suffered myocardial infarctions. So, one can risk sugarcoating things too much. But … for some, I believe that the … pale finger of death … might, in some way, help to bring about change [laughs] I believe that.

[Participant, cycle 4]

However, other findings revealed that some HCPs were concerned about using red to indicate a high level of risk, as they felt that it could be perceived as overly alarming or frightening:

And if they are curious and want to see this, it’s great to show them, but not unthinkingly or at any time – especially not when they are in the red zone, because that could overwhelm them.

[Participant, cycle 4]

##### Material to Prompt Reflection

Material to prompt reflection was developed by the researchers for the diabetes specialist nurses to use in risk communication. The material consisted of questions intended to enhance understanding of the relationship between T2D and cardiovascular risk, encourage personal reflection on risk, create a safe space for discussing emotional aspects such as fear, uncertainty, and hope for the future, and promote motivation for self-management.

During previous cycles, it became apparent that risk communication might be insufficient. This finding deepened the researchers’ understanding that diabetes specialist nurses may need additional support in their risk communication efforts. Consequently, the HCPs suggested that a risk-communication tool would be valuable in encouraging persons with T2D to share their thoughts. They also considered the reflection material to be important for communicating regarding cardiovascular risk. The participants suggested that the reflection material should be discussed during consultations to capture emotional responses to risk:

It sounds wiser that they receive it together with the nurse so that they can ask questions directly, instead of taking home red values and then losing all motivation, thinking that the visit wasn’t enjoyable at all, and deciding to just ignore it all.

[Participant, cycle 4]

##### Web Education for Diabetes Specialist Nurses

The results from previous cycles revealed that the diabetes specialist nurses need to recognize the importance of adopting a more person-centered approach, actively exploring and engaging with patients’ own perceptions of risk. This includes asking questions that prompt reflection on how patients perceive their personal risk and emotional aspects, such as how they feel and whether they experience anxiety when discussing potential risks. To support diabetes specialist nurses in risk communication, web education was developed.

The web education aims to improve person-centered risk communication. The content includes the description and use of supportive tools, such as a patient handbook and a risk assessment tool developed by the researchers through a co-creation process. The web education also encourages critical reflection on the role of HCPs in risk communication and provides links to relevant guidelines and scientific literature. It also incorporates visual illustrations that show how to communicate risk in a person-centered way during consultations.

During cycle 4, the HCPs felt that the web education was highly supportive for diabetes specialist nurses, meeting their needs because it can be used in a time-efficient manner. It was perceived as flexible, allowing the diabetes specialist nurses to complete each module at their own pace and suit their schedules, making it easier to integrate the training with work commitments. As one physician explained:

I think that’s fantastic, to get diabetes nurses to not feel that it’s burdensome, but to feel that they can get help and be inspired. I believe that can make an incredible difference and help them not feel alone.

[Participant, cycle four]

## Discussion

### Principal Findings

The co-creation research design was intended to give the participants a strong voice, with the researchers facilitating the participants during the workshops. This aligns with previous research showing that participants actively shape the research, rather than merely contributing insights, and that creativity and innovative thinking are central to co-creation [24]. Co-creation also strengthens trust and relationships between researchers and participants [24]. This trust is not only a prerequisite for successful collaboration but an outcome of the process. When participants feel heard and respected, relationships between researchers and participants are strengthened, and different perspectives are presented. Hence, new ideas and solutions often emerge that may not have been possible in a more hierarchical research setup.

This co-creation study involved persons with T2D and HCPs. One review has highlighted that co-creation is often used to enhance health outcomes [25]. Our co-creation process revealed insufficient person-centered risk communication. The workshops provided insights into needs, barriers, and co-creative solutions and content for interventions, such as a personal handbook, a risk assessment tool, reflection materials, and web-based education for diabetes specialist nurses. These risk communication tools are designed to drive behavior change in line with self-determination theory [26]. This co-creation intervention supports person-centered communication and self-management to reduce cardiovascular risk in T2D and has the potential to increase risk awareness in persons with T2D.

In this study, we differentiate between PAR and design thinking. Although both approaches emphasize participation, empathy, and practice-oriented knowledge development, their underlying views of knowledge, understandings of power, and goals for change differ [27]. PAR can be seen as a transformative knowledge practice because it changes relationships and structures, whereas design thinking is an innovation-focused design practice that aims to improve solutions within existing frameworks [27]. The key difference between the two lies not in the level of participation but in the purpose of participation and how power is understood and distributed during the process. Adopting a design thinking approach might have risked reducing the participants’ roles to those of users or test participants within a predefined problem framework.

The CFIR guided the development of an intervention due to constructs such as adaptability, patient needs and resources, and knowledge and beliefs about the innovation [28]. The insights helped develop strategies and materials to improve risk communication. In the “redefine and retest” phase, these were turned into a patient handbook, web-based educational resources, a risk assessment tool, and revised reflection materials. Overall, co-creation consistently guided the intervention to align with CFIR constructs (Multimedia Appendix 2).

Taking the person’s voice into account showed that language barriers and overly medicalized terminology may hinder the understanding of cardiovascular risk in T2D. However, some research suggests that HCPs prefer using medical risk tools in diabetes communication [29]. This highlights the need to balance factual communication and empathetic understanding. HCPs must recognize that both cognitive and emotional aspects influence the willingness of patients to engage with risk communication. Some persons with T2D are unaware that diabetes can lead to myocardial infarction or stroke, highlighting the need for improved risk awareness. Additionally, those living with both T2D and cardiovascular disease as a multimorbidity do not perceive the conditions to be connected and view them as 2 separate conditions [30]. This underscores the importance of developing risk communication to support persons with T2D in gaining a deeper understanding of the seriousness of their condition and improving awareness of the crucial role that self-management plays in mitigating cardiovascular risk in T2D. It is crucial to communicate risk to patients in relation to their own perspective on their disease and perceived risk, as this approach is essential to enhancing person-centered risk communication and supporting effective self-management.

Problem-solving and idea generation during the workshops revealed valuable insights to support person-centered approaches in risk communication consultations. The findings highlighted the need for HCPs to actively explore the emotional aspects of T2D and to use individualized, verbal, and written communication that is more direct, less vague, and accessible, so that it is easier for persons with T2D to understand and refer to. Persons with T2D emphasized the importance of undivided attention, emotional presence, and meaningful dialogue. Research shows that stigma associated with T2D can negatively affect the communication efforts of HCPs by, for example, their attributing blame and pointing to personal shortcomings such as a lack of willpower, discipline, or motivation to improve one’s health. This can lead to negative perceptions of persons with T2D, as well as reduced confidence in their ability to communicate in a person-centered manner, and less effective motivational conversations [31]. A person-centered approach has been shown to reduce diabetes-related stigma, which in turn can facilitate improved risk communication [32].

This study demonstrated that co-creation can be used to develop a wide range of prototypes and interventions. The workshops gave persons with T2D and HCPs an active role in shaping an intervention, rather than being passive recipients of it. The knowledge of the participants regarding communication of cardiovascular risk was improved throughout the workshops, aligning with previous research that suggested that knowledge fosters engagement and creativity [33]. The co-creation process emphasized shared understanding and learning, with participant feedback being crucial to the development of the intervention. The focus was on developing the intervention *with*, rather than *for*, persons with T2D and HCPs. Additionally, the findings of this research support the conclusions of other studies: involving end users in intervention development increases the likelihood of successful implementation of the intervention in clinical practice [16].

In the redefine and retest stages, the participants reviewed the proposed intervention and suggested improvements. Some HCPs were hesitant to use emotionally charged colors such as red, fearing that these might alarm persons with T2D or be perceived as aggressive. In contrast, patients expressed a desire for clearer, more direct risk communication; for example, in workshops 1 and 2, persons with T2D expressed that they want to know their personal CVD risk, which is also illustrated in the study of Jutterström [17].

Although a cautious approach to communication is important, excessive caution may hinder effective risk communication, thereby affecting patient understanding and engagement. The results also indicate a need for deeper exploration of emotions during consultations, an aspect that is often overlooked in traditional health care [34]. The developed intervention may support diabetes specialist nurses in communicating the emotional and existential aspects of T2D and cardiovascular risk more clearly [25]. Greater attention to emotional dimensions may help integrate medical knowledge with emotional well-being, creating opportunities for more person-centered communication [34]. To address this, we developed a web-based educational program for diabetes specialist nurses that promotes person-centered risk communication, encouraging patients to articulate their personal perceptions of risk. Furthermore, the risk assessment document will be tested in a pilot study. Health care professionals will be informed that they may refrain from using the instrument if they judge it to be inappropriate for a particular patient.

Future research will test and evaluate the feasibility of the intervention in a pilot study conducted in primary health care. This aligns with findings that emphasize the need for health care interventions to fit the specific structures and resources of the health care systems within which they are to be utilized [9]. Integrating the intervention into existing care pathways could enhance accessibility and sustainability, making it a practical tool for supporting risk communication.

### Study Limitations

Conducting separate workshops for patients and health care professionals prevented direct dialogue and limited insights into power dynamics and interactions. The results of previous workshops were based on earlier discussions that enabled participants to share their ideas. Additionally, our aim was to prevent a potential power imbalance [21]. It is uncertain whether the patients’ views would have been fully represented in combined workshops.

Despite the small number of HCPs involved, the workshops enabled reflective and personal discussions, fostering trust and producing rich data. Small groups allowed the participants to express and develop their ideas freely. Reflexive thematic analysis, which does not require data saturation, supports the value of such rich, qualitative input, even from limited samples [23]. However, the small and potentially homogeneous group could limit transferability [35].

A key strength was the iterative co-creation process, in which participant feedback from earlier workshops was continuously used to refine the intervention, ensuring that real-world needs were met. A potential bias may have occurred as some participants were particularly familiar or comfortable with the topic, which could have influenced the depth or direction of discussions. Recruitment challenges led to a shift from purposeful to convenience sampling, possibly reducing sample diversity. This convenience-based method may have introduced selection bias, potentially favoring participants who were already more engaged in their care. However, this can be seen as a strength, as it aims to develop and improve diabetes care.

A limitation is that the developed person-centered intervention may be more suitable for patients who can engage with written information and reflective discussions. It may be less suitable for those with language or cognitive barriers, or with limited capacity to engage with written and reflective material.

In-person workshops with persons with T2D supported empathetic, in-depth dialogue, while digital workshops suited time-constrained HCPs and enabled broader participation. Separate groups minimized power imbalances and encouraged openness, especially among persons with T2D. However, the different formats made comparisons difficult and probably prevented direct dialogue, which could have fostered mutual understanding and integration. When comparing the 4 workshops, the face-to-face sessions encouraged more spontaneous dialogue and the opportunity to meet other patients with T2D. In contrast, the digitally facilitated sessions were more practical and made participation easier for HCPs. Despite these differences, the feedback revealed similar discussions, suggesting that the results are comparable across different formats.

Through the PAR framework, the researchers positioned the persons with T2D and HCPs as active co-creators. Together, these participants developed an intervention that was intended to increase risk awareness among persons with T2D and provide them with greater control over their health. This may eventually lead to more sustainable and positive outcomes in their disease management. However, while PAR enhances relevance and collaboration, it is time- and resource-intensive, posing challenges related to tight timelines and funding constraints [13].

### Conclusions

Using a PAR framework, this study highlighted the value of co-creation in developing an intervention aiming to enhance cardiovascular risk awareness among persons with T2D. The results showed that diabetes specialist nurses need to be encouraged to explore patients’ perceptions of risk and create space to discuss emotional responses to risk during consultations. The co-created intervention consists of several components: a patient handbook, which supports patients in exploring their personal understanding of cardiovascular risk; a risk assessment tool, which visualizes individual risk factors based on laboratory values and facilitates discussions regarding risk during diabetes consultations; materials to prompt reflections to explore personal understanding of cardiovascular risk; and a web education for diabetes specialist nurses, which is designed to strengthen the person-centered approach. Web education is intended to encourage active exploration of patients’ perceptions of risk, including the emotional and existential dimensions of risk, and to guide the use of various risk-communication tools during patient encounters. The developed intervention provides a practical example of how HCPs can implement person-centered communication about cardiovascular risk in persons with T2D. By integrating the intervention in diabetes care, a person-centered approach could be facilitated, and individuals will be supported in identifying intrinsic motivation for lifestyle changes. This co-created intervention has the potential to increase risk awareness among persons with T2D.

## Supplementary material

10.2196/85748 Multimedia Appendix 1 Example of the analysis process.

10.2196/85748 Multimedia Appendix 2 The Consolidated Framework for Implementation Research (CFIR) and its relation to the study results.

10.2196/85748 Checklist 1 RTARG checklist.

## Abbreviations

CFIR: Consolidated Framework for Implementation Research
HbA_1c_: hemoglobin A_1c_
HCP: health care professional
PAR: participatory action research
T2D: type 2 diabetes
