# Supplementary material for: The Process of Developing an Intervention to Increase Awareness of Cardiovascular Risk for Persons With Type 2 Diabetes: Co-Creation Study
Source: JMIR Diabetes. 2026 Apr 23;11:e85748. doi: 10.2196/85748 (PMC13105426; doi:10.2196/85748)
Supplement: Multimedia Appendix 1 [file diabetes-v11-e85748-s001.docx]

Appendix 1 Example of the analysis process

| Meaning units | Codes | Subthemes | Themes |
| --- | --- | --- | --- |
| I don't just want the diabetes nurse to check my values. I want her to show curiosity and care about me, asking how I'm feeling and demonstrating her commitment to me. | To make me feel like you care only about me right now | Desiring to be cared for | Co-define  Taking the patient's voice into account |
| My diabetes nurse is very good at explaining things in a simple way. Instead of getting fixated on exact values and figures, I just want to know: Is it good or bad? And if it's bad, what should I do about it? | To clarify the individual risk in a risk profile | Ideas for enhancing risk-communication | Co- design  Problem-solving and generating ideas |
