# Supplementary material for: The Process of Developing an Intervention to Increase Awareness of Cardiovascular Risk for Persons With Type 2 Diabetes: Co-Creation Study
Source: JMIR Diabetes. 2026 Apr 23;11:e85748. doi: 10.2196/85748 (PMC13105426; doi:10.2196/85748)
Supplement: Multimedia Appendix 2 [file diabetes-v11-e85748-s002.docx]

| Theme | Subtheme | Participatory Action Research (PAR) framework | Developed Intervention Component | CFIR Construct | CFIR Domain |
| --- | --- | --- | --- | --- | --- |
| Co-define:  Taking the patient's voice into account | Desiring to understand and to be understood | Defining the problem/Identifying barriers/needs | Need for clear and accessible language in risk communication | Adaptability | Innovation |
|  | Desiring not to be left out and deprioritised | Defining the problem/Identifying barriers/needs | Need for HCPs curiosity, interest, and engagement | Patient Needs & Resources | Outer Setting |
|  | Desire to be cared for | Defining the problem/Identifying barriers/needs | Need for trust and mutual understanding during consultations | Knowledge & Beliefs about the Innovation | Characteristics of Individuals |
| Co-design:  Problem-solving and generating ideas | Ideas for enhancing risk communication | Problem-solving | Strategies for improved risk communication | Design Quality & Packaging | Innovation |
|  | Respectful approach and social support | Developing joint Solutions | Communication prompts and supportive materials | Source | Innovation |
| Prototype and test drafting intervention proposals | Proposals on risk assessment tools | Drafting intervention proposals | Prototype of risk-assessment tool | Evidence Strength & Quality | Innovation |
|  | Proposals to support HCPs in risk communication | Drafting intervention proposals | Educational material for person-centred risk communication | Reflecting & Evaluating | Process |
|  | Proposals to support persons with T2D | Drafting intervention proposals | Patient handbook and reflective materials to support reflection and exploration of cardiovascular risk. | Adaptability | Innovation |
| Re-define and re-test: reviewing suggested interventions | Patient handbook | Reviewing the suggested interventions | Refined patient handbook | Reflecting & Evaluating | Process |
|  | Web education for diabetes specialist nurses | Reviewing the suggested interventions | Web education for diabetes specialist nurses | Access to Knowledge & Information | Inner Setting |
|  | Risk assessment tool | Reviewing the suggested interventions | Finalised risk assessment tool aligned with clinical guidelines | External Policy & Incentives | Outer Setting |
|  | Material to prompt reflection | Reviewing the suggested interventions | Adjusted materials to strengthen professional ownership | Knowledge & Beliefs about the Innovation | Characteristics of Individuals |
