# Supplementary material for: The Process of Developing an Intervention to Increase Awareness of Cardiovascular Risk for Persons With Type 2 Diabetes: Co-Creation Study
Source: JMIR Diabetes. 2026 Apr 23;11:e85748. doi: 10.2196/85748 (PMC13105426; doi:10.2196/85748)
Supplement: Checklist 1 [file diabetes-v11-e85748-s003.docx]

**Author Evaluation Tool Using the Reflexive Thematic Analysis Reporting Guidelines (RTARG)**

| **Advice for approach to reporting** | **Author comment / justification** |
| --- | --- |
| **Background and rationale** | |
| Provide a robust context and rationale for the proposed research in the introduction | The introduction outlines issues and gaps in current literature and highlights the partial perspective of qualitative research in relation to the research problem. |
| Clearly articulate a research question – one that is methodologically coherent | At the end of the introduction, the aim of the study is clearly outlined. |
| **“Owning your perspectives”** | |
| Include information on guiding theoretical assumptions and other (e.g., explanatory) theory informing the use of TA | The researchers emphasise the importance of adopting a co-creation approach to understanding the participants' experiences, keeping in mind that each participant is unique and subjective. C**onceptual coherence** ensures that the theoretical assumptions, research questions, data collection methods, and type of TA used all align. |
| Report in a way that is consistent with stated theoretical assumptions throughout | The analysis was conducted using reflexive thematic analysis, informed by a social constructionist perspective. Participants’ experiences were interpreted as shaped by sociocultural contexts, and the researcher’s role was acknowledged as active and interpretive. Conceptual coherence was maintained by aligning theoretical assumptions, research questions, data collection, and analytic approach. |
| Evidence methodological coherence/integrity in both the research and the report | The study followed Braun and Clarkes (2022) approach to RTA in both data generation and analysis to ensure that the process was structured yet flexible. Justification of why, how and when the approach was used is also discussed in the report. This evaluative tool created based on the RTARG has also provided a framework to ensure rigor, transparency and credibility in the analysis and reporting process. |
| Show evidence of reflexive practice | The methodology provides a ‘discussion of reflexivity’ section which notes the importance of all authors recognising possible unconscious bias due to their professional identities. |
| Write in a methodologically coherent style | The justification for why a qualitative Co-creation process within a PAR framework was chosen is discussed in the introduction and methodology. The paper also adhered to guidance from Braun and Clark's (2022) approach to RTA from data collection, analysis, and through to the report. All authors acknowledged the role of reflexivity and discussed how their personal interpretations could cause potential bias. |
| **Participants/data items** | |
| Describe the selection of participants/data items | How participants were recruited, inclusion/exclusion criteria were used to describe the selection of participants in the participants and settings section. |
| Provide a rational or explanation around data set or participant group size/composition | The ‘participants and setting section of methodology explains how the study, as guided by Braun and Clarke (2022) review on data saturation, focused on ensuring the quality and depth of data rather than excessively prioritising data saturation. |
| Discuss characteristics of participants/data items | The number of participants in every workshop, age, and working years in primary health care were used to provide an overview of participants. This information offers a general understanding of the sample's demographic and clinical profile without revealing individual identities. Demographic information on the participants is presented in a table. |
| Detail ethical approval and ethical code/principles followed, participants' informed consent, etc | The methodology section detailed that Ethical approval was granted by the Swedish Ethical Review Authority and noted that participants were informed verbally and in writing about the study’s aim, participation, the right to withdraw, and confidentiality. |
| **Dataset generation** | |
| Provide some rationale for the method(s) for data generation/data item sources chosen. | RTA, the chosen methodological approach, was justified in the methodology of the manuscript as to why the technique was deemed best suited. The rationale for how methodology was applied to dataset generation and analysis is also addressed in the manuscript. |
| Describe the development and/ or characteristics of data generation tool(s) | A figure of the Co-creation process within a PAR framework is provided, and detail of characteristics of data generation tools, such as an audio recorder, picture presentations, worksheets, and scenario-based exercises, was also detailed in the ‘dataset generation’ section of the methodology. |
| Include details such as modality and/ or setting of data generation, time frame, and other pertinent procedural information | Details on where data generation was conducted, and the timeframe are detailed in the ‘dataset generation’ section of methodology. |
| Describe who conducted any interactive data generation (which author or research role), and how. | The ‘data analysis procedures’ section of methodology details how and when the authors interacted with the data generation. |
| Describe the size/scope of the dataset and dataset items. | The description of the dataset size, gender, age, and characteristics is shown in the ‘Participants’ and setting section of the methodology Demographic information on the participants is presented in a table. |
| Describe, and if relevant, explain any preparation of data for analysis. | The ‘Dataset generation’ section of the methodology described how audio recordings from encounters with participants were transcribed to make raw data files of each workshop before analysis took place. |
| **Data analysis** | |
| Provide some rationale for use of RTA, and, where relevant, for combining RTA with other approaches and procedures | The ‘Design section’ of methodology provides reason as why RTA was decided as the best technique for the study. |
| Discuss how the researcher(s) engaged with the analytic process | The ‘Data analysis procedures’ section of methodology provides a step-by-step guide on how the author (ALS) initially generated codes. The generated themes and created themes were made by (AL. LJ och KHÄ). These were then then discussed and explored by author (KHÄ and LJ ) before being examined and reviewed by the rest of the team (Al, LJ, KHÄ, JO, ML). |
| Where more than one person is involved, describe who analysed the data (author or research role). | Authors were referred to by their initials (e.g. ALS) to describe who analysed the data and at what point. All researchers’ roles were detailed in the ‘researcher description and discussion of reflexivity’ section of the methodology. |
| Use language to describe the process and products of RTA that is coherent with the values and assumptions of RTA. | Author ALS dedicated time to understanding the values of RTA by reviewing relevant literature and guidance from Braun and Clarke. All authors were mindful to ensure that both the process and the outcomes were flexible and reflexive. Self-reflection was actively practised throughout the research, particularly after engaging with the data. The researchers acknowledged their interpretative role and emphasised the importance of triangulation to mitigate any potential bias. |
| **Reporting the data analysis** | |
| Provide an overview of themes or thematic structure | The ‘findings' section describes all themes and subthemes. |
| Ensure theme conceptualisation is appropriate to RTA, and any divergences are justified and explained. | The themes were not predefined or rigidly applied but instead generated interactively through reflective engagement with the data. All authors examined, explored and agreed on all theme names. |
| Name themes appropriately | All authors explored and examined possible theme names, and after some reflection, names were agreed on collectively. |
| Report themes in sufficient depth and detail. | The ‘analysis’ section of the report details an explanation as to why the theme and subtheme names were chosen, and the relationship between them, and that the analysis followed the steps outlined by Braun and Clarke (2022).  The narrative was designed to illustrate the meaning of the data and explain its relevance, ensuring the findings were interpreted to highlight their broader implications. Paraphrasing and marginal notes were incorporated to preserve contextual relevance and support the analytical process |
| Use subtheme judiciously | Subthemes were thoughtfully selected and only chosen when all the group agreed they were relevant to the main themes and contributed meaningfully to the overall analysis. |
| Ensure the analytic narrative explains the meaning and significance of the data. | All researchers felt it was important that the narrative was designed to illustrate the meaning behind the data and explain its relevance, ensuring the findings highlight their broader implications. Thus, three of the researchers (ALS, LJ, and KHÄ) were part of creating the analytical narrative, and conversations on what these interpretations mean were conducted throughout the write-up period. |
| Provide an appropriate balance of analytic narrative and data extracts – both data extracts and analytic narrative matter. | Following Braun and Clarkes' (2022) guidance, the analysis integrated a balance between deep discussion of interpretation and direct extracts as evidence within each subtheme. |
| Demonstrate coherence between analytic narrative and illustrative/evidentiary data extracts. | The analysis followed a structured approach by which an analytical narrative was always used to introduce and explain a theme and subtheme. A direct extract was then used to provide evidence, showing how the personal experiences align with the analytical interpretation. |
| Integrate existing research and theory into the analytic narrative. | The background section presents how the self-determination theory (SDT) and the Consolidated Framework for Implementation Research (CFIR) are integrated into our research. The 'discussion' section of the report referenced relevant literature to both support and, at times, challenge the themes identified in the data. For instance, the Participatory Action Research (PAR) framework aligned with the current study's findings. We have clarified in the manuscript and a table how our results and the PAR process align with CFIR constructs. |
| **Quality, evaluation, and conclusions** | |
| Draw analytic conclusions across themes. | The ‘discussion’ section provides an analytical conclusion on the themes and their significance, comparing them with previous literature. |
| Discuss implications or directions for future research | Using the analysis, the ‘clinical implications’ section of the report makes future research and intervention recommendations for the patient group and the healthcare professionals in primary health care. |
| Use and report quality practices coherent with RTA. | The RTARG was used to create an evaluative tool to critically appraise the present study for methodological congruence and reflexive openness. |
| Evaluate the research from a Big Q standpoint. | The study aimed to explore, understand, and interpret the in-depth lived experiences of those with type 2 diabetes and the health care professionals while adhering to the principles and rigor of the chosen qualitative approach. In keeping with a Big Q qualitative approach, patient and co-creation were also central to ensuring the research was valid, contextually grounded, and socially meaningful. The Co-creation design supported a reflexive process and helped align the study with the lived realities of those it aimed to represent. Overall, the researchers feel the study provided a meaningful contribution to the phenomenon under study, whilst also recognising how their roles might shape the intervention. |
| Include reflections on the research process and practices, including researcher reflexivity. | The ‘researcher description and discussion of reflexivity’ section of the manuscript acknowledges prejudices, biases, and assumptions that may occur in the research, and the manuscript also provides a ‘strengths and limitations’ section where the researchers reflected on the research process and practices. |
